# A Case of Interrupted Aortic Arch in an Adult: An Unsuspected Cause of Uncontrolled Hypertension

**DOI:** 10.1155/2024/8691643

**Published:** 2024-04-20

**Authors:** Abdi Dandena Dibaba, Abenezer Kebede Bekele, Selam Hagos Gebrewahd

**Affiliations:** ^1^Department of Radiology, Faculty of Medical Science, Institute of Health, Jimma University, Jimma, Ethiopia; ^2^Department of Radiology, Soddo Christian General Hospital, Soddo, Ethiopia; ^3^Pediatrics Unit, Ethiopian Armed Force Comprehensive Hospital, Addis Ababa, Ethiopia

## Abstract

In this paper, we describe an incidentally discovered case of interrupted aortic arch in a 28-year-old male patient with a history of long-standing poorly controlled hypertension. The patient presented to the hospital with a complaint of upper respiratory complaints and long-standing chest pain. A plain chest radiograph was requested to exclude a diagnosis of pneumonia, and the radiography spotted an incidental finding of inferior rib notching. A subsequent CT angiography was done for further characterization, and a diagnosis of interrupted aortic arch was confirmed. Therefore, although rare, IAA should be considered in adults with refractory hypertension or unexplained congestive heart failure.

## 1. Introduction

Interrupted aortic arch (IAA) is a rare congenital malformation of the aorta and aortic arch. It is defined as a lack of luminal continuity between the ascending and descending thoracic aorta. IAA is an extremely rare condition that represents less than 1% of all congenital heart diseases occurring in 3 per million live births [1].

This rare find was initially described in the surgical literature back in 1778 by Stiedele RJ and was later classified into three subtypes by Celoria and Patton in 1959 based on the location of the atretic segment: discontinuity distal to the left subclavian artery is classified as type A, discontinuity between the left common carotid and the left subclavian artery is type B, and discontinuity between the innominate and left common carotid artery is classified as type C [2].

## 2. Case Presentation

A 28-year-old male patient presented to the hospital outpatient department with a complaint of a history of cough, chest pain, and upper respiratory tract symptoms including runny nose, sore throat, and cough. Further clinical history revealed that patient has had systemic hypertension for the past 13 years being followed at different healthcare facilities. The patient is taking three antihypertensive medications for his blood pressure (almondine 10 mg daily, hydrochlorothiazide 25, and enalapril 10 mg BID).

**Figure 1:**
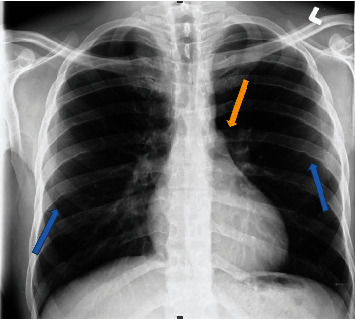
Poster anterior (PA) chest radiograph of the patient showing bilateral irregularities in the inferior aspect of the ribs suggestive of inferior rib notching (blue arrow) and absence of the aortic knuckle (orange arrow).

The patient was investigated with a complete blood count (CBC) and chest radiography with a clinical suspicion of pneumonia. The CBC findings showed lymphocytosis suggestive of a viral infection. The CXR showed normal lung fields with no signs of pneumonia. On further examination of the osseous structures in the chest, there were multiple subtle irregularities involving the bilateral inferior ribs suggestive of inferior rib notching (Figure 1).

Following the chest X-ray findings, the patient was re-evaluated with a clinical suspicion of coarctation of the aorta. The blood pressure from the upper and lower extremities was taken and showed significant discrepancy with the upper extremities registering a blood pressure of 150/100 mmHg and the lower extremities registering a blood pressure of 90/65 mmHg. The pulses in the lower extremities at the popliteal arteries and dorsalis pedis were feeble compared to the pulses felt in the bilateral radial arteries.

**Figure 2:**
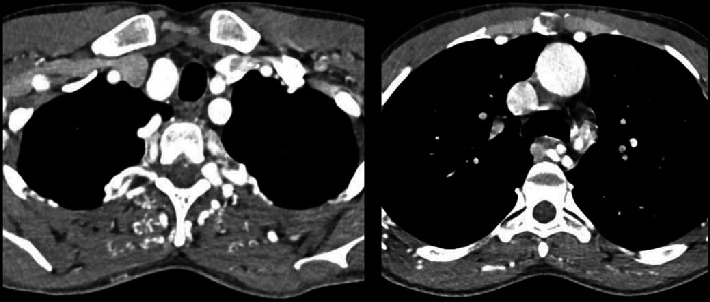
Axial postcontrast CT scan images at the level of the upper thoracic cavity, showing multiple tortuously enlarged collateral vessels in the chest wall and mediastinum.

**Figure 3:**
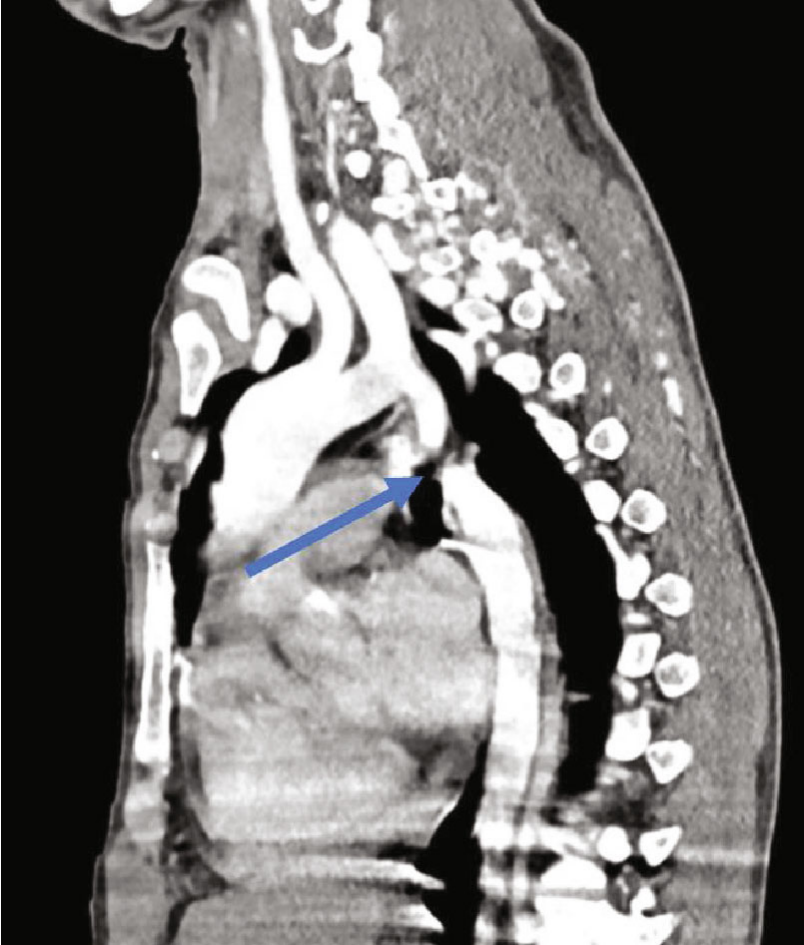
Sagittal reconstructed image of the CT angiography showing a complete discontinuity between the aortic arch and the descending thoracic aorta (arrow).

**Figure 4:**
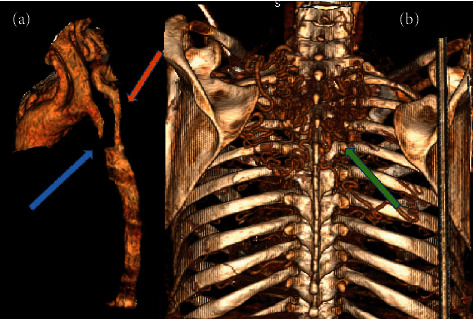
(a) 3D volume rendered images showing a complete discontinuity between the aortic arch and the descending thoracic aorta (blue arrow). (b) 3D volume rendered images of the collateral vessels seen in the thoracic wall (green arrow) connecting to the postatretic segment (orange arrow).

The patient was subsequently investigated with a computed tomography angiography (CT-A) which showed large collateral vessels all over the chest wall with tortuously dilated internal mammary arteries, posterior and anterior intercostal arteries, and large paravertebral and parascapular collateral vessels (Figure 2). On the sagittal reconstructed images, there is a complete discontinuity between the aortic arch and descending thoracic aorta which was a 1.5 cm gap (Figure 3) suggestive of a type A interrupted aortic arch. A reconstruction in 3D of the defect vividly displays the distinct outline of the aorta interruption, with only collateral vessels supplying flow to the distal segment (Figure 4).

The patient was counseled on his condition and treatment options and decided to defer surgery and continue with conservative medical treatment.

## 3. Discussion

Interrupted aortic arch is among the rare causes of secondary hypertension and is often delayed in the diagnosis. This case demonstrates a complete disruption of the aortic arch and the descending thoracic aorta in a patient who has been treated for hypertension for the past 13 years. In general, complete discontinuity of the aortic arch with no flow towards the distal vessels is often fatal. Hence, IAA is not usually suspected as a cause of secondary hypertension in an adult patient. However, patients with isolated IAA may survive until adulthood if extensive collateral vessels are joining the descending aorta.

The normal thoracic aorta is formed by the creation of six bilateral aortic arches and the seventh intersegmental arteries, with the involution of various segments of these. A type A IAA is thus formed by the abnormal regression of the left fourth arch segment late in development after the left subclavian artery has ascended to its normal position. A type B IAA is found when the left fourth arch segment regresses early, before cephalad migration of the left subclavian artery. A type C IAA seems to represent involution of the ventral portion of the left third arch and the left fourth arch (both of which arise from the left limb of the aortic sac) and persistence of the normally regressing ductus cautious [3].

Presentation of IAA differs between adulthood and infancy. Neonates are likely to present with signs of heart failure, and it is commonly associated with other congenital malformations such as patent ductus arteriosus, truncus arteriosus, ventricular septal defects, and aorticopulmonary window. And if it is left untreated surgically, most infants will succumb within the first week of life with a median age of 4 days. This is mostly due to greatly increased left-to-right shunt with increased pulmonary blood flow, which results in biventricular failure, pulmonary edema, ductal stenosis, and its sequelae, renal failure, and metabolic acidosis [1].

IAA seen in adults is different from that of infancy, in a study that reviewed around thirty-plus cases. Type A was the most common type accounting for 79% of the cases while type B was the most common subtype during infancy which accounted for around 53% of the cases. The average age presentation was around 39.4 years, with patients ranging from 18 to 72 years [4].

IAA can be associated with other congenital malformations, and IAA is commonly linked with the DiGeorge syndrome. The DiGeorge syndrome is a rare condition that results in thymic hypoplasia or aplasia and is associated with different cardiac anomalies, developmental delay, and abnormal facies. 50% of patients with interrupted aortic arch carry the gene mutation responsible for the DiGeorge syndrome, a deletion in the gene 22a11.2. Additionally, other syndromes such as CHARGE syndrome have been associated with IAA [5, 6].

The clinical presentation is often subtle and may be asymptomatic. The common clinical symptoms and signs may be a headache, chest pain, and claudication. Hypertension refractory to conventional treatments, differences in upper and lower limb blood pressure, limb swelling, congestive heart failure, or aortic dissection can also be suggestive features of IAA in an adult patient [5].

The diagnosis of IAA is often delayed and often not clinically unsuspected. Therefore, imaging is very critical in making the diagnosis and proper surgical planning. Most often routine imaging modalities such as chest X-rays may be normal or may show subtle cardiomegaly and/or rib notching which can often be missed [6].

Echocardiography is among the important primary imaging modalities used in the investigations of the anomalies of the heart and the great vessels. Echocardiography has the advantage of being portable, noninvasive, and lack of ionizing radiation. Studies have shown that echocardiography can be used to give an accurate preoperative diagnosis in children with interrupted aortic arch [7].

Catheter angiography was once commonly used for the evaluation of the patient suspected of IAA. Despite its high temporal resolution and real-time imaging, it has largely been replaced by less invasive diagnostic modalities such as CT and MR angiography. Currently, the use of catheter angiography is mainly reserved for patients who are candidates for percutaneous interventions [8].

The advent of newer imaging modalities such as CT and MR angiography has revolutionized the diagnosis of IAA. CT and MR angiography are valuable noninvasive methods employed for morphologically assessing aortic discontinuities. They are frequently adequate for preoperative planning purposes [9].

CT angiography has the advantage of being more accessible and having a high temporal and spatial resolution compared to other imaging modalities. Additionally, the quick scanning time is particularly useful in trying to assess children without the use of sedation [10].

MRI has emerged to be useful in the evaluation of various congenital cardiac and vascular malformations in children and adults and has the advantage of being noninvasive and does not require ionizing radiation which is a concern in the pediatric population. The major drawback of MRA is that it may require sedation or general anesthesia to limit motion-related artifacts in children [11].

The treatment of IAA has come a long way through the years with Van Praagh et al. describing IAA as the most rapidly lethal form of congenital cardiovascular disease which remained an incompletely solved therapeutic problem in the 1970s [12]. Currently, the main treatment for IAA is surgical intervention or percutaneous stent placement, with the choice of surgical technique depending on the age of the child affected for neonates and infants, several surgical means, such as end-to-end or end-to-side anastomosis, exist. While older children and adults can undergo end-to-end anastomosis, the older population can undergo placement of interposition grafts [13].

In the review of the literature on the different treatment options and outcomes, surgical correction was performed in nearly 81% of the cases and around 8% of patients received percutaneous wire perforation and stenting through the septum dividing the ascending and descending aorta. The rest (10%) were managed medically after refusal of surgical management [4].

Surgery for correction of IAA is successful in most patients with resolution of symptoms such as claudication and paresthesia following the procedure, and the elevated blood pressure also subsides gradually, and antihypertensive medications can be discontinued [14].

## 4. Conclusion

IAA is an often unsuspected cause of uncontrolled hypertension, and although it is rare, it must be considered along with aortic coarctation in the differential diagnosis of an adult patient with a refractory hypertension nonresponsive to medical management. Careful physical examination of the lower and upper peripheral pulses in adults with a chief complaint of hypertension is of utmost importance for suspecting IAA as a potential cause. CT angiography is an important tool for noninvasive imaging modality for making the diagnosis of the IAA and also for guiding surgical planning by mapping the location of the discontinuity and the collateral vessels.

## Data Availability

The Full dicom images used to support the findings of this case report are available from the corresponding author upon request.

## Acronyms

CBC: Complete blood count
OPD: Outpatient department
CT- A: Computed tomography angiography
MRI: Magnetic resonance imaging
MRA: Magnetic resonance angiography
IAA: Interrupted aortic arch.
